# Dietary Patterns Derived by Cluster Analysis are Associated with Cognitive Function among Korean Older Adults

**DOI:** 10.3390/nu7064154

**Published:** 2015-05-29

**Authors:** Jihye Kim, Areum Yu, Bo Youl Choi, Jung Hyun Nam, Mi Kyung Kim, Dong Hoon Oh, Yoon Jung Yang

**Affiliations:** 1 Department of clinical nutrition, Graduate School of Health Sciences, Dongduk Women’s University, 23-1 Wolgok-dong, Sungbuk-gu, Seoul 136-714, Korea; E-Mails: eluzai81@gmail.com (J.K.); arreumnew@naver.com (A.Y.); 2 Department of Preventive Medicine, College of Medicine, Hanyang University, 17 Haengdang Dong, Sungdong Gu, Seoul 133-791, Korea; E-Mails: bychoi@hanyang.ac.kr (B.Y.C.); kmkkim@hanyang.ac.kr (M.K.K.); 3 Department of Psychiatry, College of Medicine, Hanyang University, 17 Haengdang Dong, Sungdong Gu, Seoul 133-791, Korea; E-Mails: jhnama@hanyang.ac.kr (J.H.N.); odh@hanyang.ac.kr (D.H.O.); 4 Department of Foods and Nutrition, College of Natural Sciences, Dongduk Women’s University, 23-1 Wolgok-dong, Sungbuk-gu, Seoul 136-714, Korea

**Keywords:** dietary pattern, older adults, cluster analysis, cognitive impairment

## Abstract

The objective of this study was to investigate major dietary patterns among older Korean adults through cluster analysis and to determine an association between dietary patterns and cognitive function. This is a cross-sectional study. The data from the Korean Multi-Rural Communities Cohort Study was used. Participants included 765 participants aged 60 years and over. A quantitative food frequency questionnaire with 106 items was used to investigate dietary intake. The Korean version of the MMSE-KC (Mini-Mental Status Examination–Korean version) was used to assess cognitive function. Two major dietary patterns were identified using K-means cluster analysis. The “MFDF” dietary pattern indicated high consumption of Multigrain rice, Fish, Dairy products, Fruits and fruit juices, while the “WNC” dietary pattern referred to higher intakes of White rice, Noodles, and Coffee. Means of the total MMSE-KC and orientation score of the participants in the MFDF dietary pattern were higher than those of the WNC dietary pattern. Compared with the WNC dietary pattern, the MFDF dietary pattern showed a lower risk of cognitive impairment after adjusting for covariates (OR 0.64, 95% CI 0.44–0.94). The MFDF dietary pattern, with high consumption of multigrain rice, fish, dairy products, and fruits may be related to better cognition among Korean older adults.

## 1. Introduction

The proportion of the world’s population aged ≥60 years was 12% in 2013 and is expected to reach 21% by 2050 [1]. Along with the increase in the aging population, the prevalence of dementia has also been increasing rapidly [2], and cost for the care of dementia will also be increased with its prevalence [3]. In 2011, the cost of caring for dementia alone was approximately US $7.1 billion in Korea, and it was estimated that this would increase to about US $100.6 billion by 2050 [3]. Therefore, the social and economic burden is expected to increase significantly.

Mild cognitive impairment (MCI) can be defined as an intermediate state of cognitive function between cognitive decline, seen in normal aging and dementia [4]. Several studies reported MCI to be a great risk factor for developing dementia [5,6]. As a result, it is important to prevent MCI in order to reduce the risk of dementia.

Many studies have investigated the association between specific nutrients and cognitive function, but the associations of specific nutrients such as antioxidant vitamins, B-vitamins, and n-3 polyunsaturated fatty acids with cognitive function have not been consistent [7,8,9,10,11,12,13]. People consume various foods with complex combinations of nutrients. Therefore, dietary pattern analysis may be a useful tool to consider overall diet, and can be used to investigate the association between overall diet and cognitive function.

Several studies conducted in western countries have reported that “whole food” [14], “healthy” [15,16], and “Mediterranean-style” [17] dietary patterns were related to better cognitive function. In addition, “processed food” dietary patterns were related to a higher risk of cognitive impairment [14,18]. In Asian countries, a “vegetables-fruits” dietary pattern (higher intake of vegetables, fruits, soy products and legumes) and a “snacks-drinks-milk products” dietary pattern (higher intake of fast food, sweets and desserts, nuts, and milk products) were related to a lower risk of cognitive impairment in older Chinese people [19]. Koreans have distinct dietary patterns, but few studies have been conducted on the association between Korean dietary patterns and MCI. Thus, the objective of this study was to investigate major dietary patterns by using cluster analysis, and to determine an association between the dietary patterns and cognitive function among Korean older adults.

## 2. Materials and Methods

### 2.1. Study Population

The Korean Multi-Rural Communities Cohort Study (MRCohort), which is a part of the Korean Genome Epidemiology Study (KoGES), has been conducted since 2004 to determine risk factors for cardiovascular disease in the Korean population. The target population of this community-based cohort is residents aged 40 years and over living in rural areas (Yangpyeong, Gyeonggi, South Korea). Cognitive function has been assessed in the participants aged 60 years and over since 2009. Thus, the participants included in the present study were the participants aged 60 years and over for which data were collected between July 2009 and August 2010 (*n* = 808). Participants with implausible self-reports on energy intake of <500 kcal/day (*n* = 2) were excluded. Since our study is a cross-sectional design study, older people already having cognitive impairment lose their ability to prepare or purchase healthy foods, thus the possibility of reverse causality can exist. Therefore, to reduce the influence of inaccuracy conferred by participants with cognitive impairment, participants with the lowest 5% of cognitive function score (3–14; mean ± SD = 11.6 ± 2.8) were eliminated [20]. Thus, the final subjects were 765 participants aged 60 years and over (331 men and 434 women). The Declaration of Helsinki was upheld, and all procedures involving human participants were approved by the Institutional Review Board of Hanyang University. Written informed consent was obtained from all participants.

### 2.2. General Characteristics and Anthropometrics Variables

All procedures were conducted according to standardized protocols developed for examination and questionnaire procedures. A structured questionnaire was conducted by trained interviewers to obtain information on demographic characteristics (age, sex, education, and marital status), medical history (hypertension, hyperlipidemia, diabetes, cardiovascular disease, or stroke), and lifestyle factors (smoking, alcohol consumption, regular exercise, and intake of dietary supplements). Participants were asked to report whether they had smoked or had consumed alcoholic drinks in their entire life. If they had smoked or consumed alcoholic drinks in their entire life, they were additionally asked whether they were former smokers/drinkers or current smokers/drinkers. The proportions of current smokers/drinkers were presented in the results. If participants reported that they had exercised regularly, they were classified into regular exercisers. Anthropometric measurements were acquired by using standardized methods. Height was measured using a standard height scale to within 0.1 cm. Participants were weighed using a metric weight scale to the nearest 0.01 kg in light clothing without shoes. Body mass index (BMI) was calculated as weight (kg)/height (m^2^).

### 2.3. Cognitive Function Examination

The Mini-Mental State Examination (MMSE) is one of the most frequently used screening tools for the assessment of cognitive function [21]. In this study, the MMSE-KC (Mini-Mental Status Examination-Korean version) was used to assess cognitive function. The MMSE-KC was developed as a part of the Korean version of the Consortium to Establish a Registry of Alzheimer’s Disease (CERAD) Assessment, and it has been proven to be as equally valid and reliable as the English version of the CERAD [22]. The MMSE-KC was administrated by trained interviewers using a standard protocol. Scores can range from 0 to 30, with higher scores indicating better cognitive function. The MMSE-KC consists of the following areas: orientation (10 points), memory (3 points), attention and calculation (5 points), memory recall (3 points), language function (6 points), visuospatial construction (1 points), and understanding and judgment (2 point). Since MMSE is affected by socio-demographic characteristics, the participants were diagnosed as having mild cognitive impairment (MCI) using the criteria of the MMSE-KC according to age, sex, and education [23]. Participants with scores less than 1.5 standard deviations from the mean were categorized as “MCI”. Among items from the MMSE-KC, orientation and memory evaluation were used to identify a relationship between dietary patterns and cognitive function with the total score of the MMSE-KC. The MMSE-KC has been applied in several studies to assess the cognitive function of Koreans [24,25,26].

### 2.4. Dietary Data

The quantitative food frequency questionnaire (FFQ), with 106 food items, was administrated by trained interviewers to assess dietary intake. The validity and reproducibility of the FFQ have been reported in detail elsewhere [27]. Daily intakes of specific nutrients were calculated by multiplying the frequency of consumption per day, portion size of the 106 food items in grams, and nutrients per gram. Nutrients per gram were acquired using CAN-PRO 4.0 (Computer Aided Nutritional Analysis Program, the Korea Nutrition Society).

### 2.5. Statistical Analysis

The 106 food items were consolidated into 23 food groups depending on the similarity of intakes and nutrient profiles: white rice, multigrain rice, noodles, rice cakes, cereals, breads, sweet foods, nuts, beans, eggs, potatoes, salty vegetables, vegetables, meats, soups, fish, seafood, dairy products, soymilk, coffee, green tea, soft drinks, and fruits and fruit juices.

Factor analysis is commonly used to detect a dietary pattern by finding factors that are composed of correlated dietary variables. Individuals cannot be classified into distinct groups by factor analysis. Unlike factor analysis, cluster analysis classifies individuals into relatively homogeneous groups, thus, cluster analysis enables us to compare distinct groups directly. Cluster analysis was performed to derive dietary patterns and to divide participants based on the similarity of their diets using the FASTCLUS procedure. This procedure sorts participants into relatively homogeneous groups through use of the K-means method. The K-means cluster analysis, a non-hierarchical cluster technique, is done on the basis of Euclidean distances; therefore, the centers of cluster are grounded on least squares estimation. This analysis requires the number of clusters to be specified prior to analysis. The FASTCLUS procedure was initially run with 20 clusters, and then participants in clusters with fewer than 5 participants were temporarily removed [28]. From this sample, the number of clusters was varied (from 2 to 6) to determine the optimal number of clusters to provide a solution of reasonable size, with the interpretable clusters. Through this process, two cluster solutions were selected as the representation of the dietary patterns in this population. To evaluate the stability of the clusters, K-means cluster analysis was conducted repeatedly with two cluster solutions in random samples of 50% of the participants, and similar results were identified as those in the original analysis. Once the specified number of clusters was settled, the participants were reassigned to the nearest cluster [28].

Nutrient Adequacy Ratios (NAR) and Mean Adequacy Ratio (MAR) were computed for protein and micronutrients (Vitamin A, Vitamin C, thiamine, riboflavin, niacin, Vitamin B_6_, folate, Vitamin B_12_, calcium, phosphorus, magnesium, iron, and zinc) on the basis of the recommended nutritional intake (RNI) from the Dietary Reference Intakes for Koreans (KDRIs) to evaluate the nutrient adequacy of the diet depending on the dietary pattern [29]. The NAR is defined as the ratio of a certain nutrient intake to its RNI. The MAR was computed by calculating the average of the NAR values. A MAR value of 100% shows that dietary intake is equal to the RNI.

Characteristics of the participants and food intakes were compared between the dietary patterns using the Chi-square test for categorical variables and *t*-test for continuous variables, and the variables which showed significantly different distributions between the dietary patterns were considered as potential confounders. Nutrient intakes and MMSE-KC scores were tested by the general linear model compared between the dietary patterns after adjusting potential confounders (age, sex, education, alcohol consumption, regular exercise, and history of diabetes). Multivariate logistic regression analysis was conducted to investigate the association between major dietary patterns and cognitive function after adjusting for potential confounders. All statistical analyses were performed using SAS software (version 9.3; SAS Institute, Cary, NC, USA).

## 3. Results

A total of 36% of the participants were categorized into the cognitive impairment group. Two major dietary patterns were identified using the K-means cluster analysis in the study population. Group names were assigned based on the foods and food groups with high consumption. Mean intakes (g/day) of foods and food groups of the two dietary patterns are presented in Table 1. The Cluster 1 dietary pattern had higher mean intakes of Multigrain rice, Fish, Dairy products, and Fruits and fruit juices. Consequently, this pattern was named the “MFDF dietary pattern”. The Cluster 2 dietary pattern was characterized by significantly higher mean intakes of White rice, Noodles, and Coffee, for which it was named the “WNC dietary pattern”.

**Table 1:** Means of food and food groups intakes in the two dietary patterns.

Foods & food groups	MFDF (*n* = 589)	WNC (*n* = 176)	*p* ^1^
White rice (g/day)	0.2 ± 5.6	254.4 ± 78.6	<0.0001
Multigrain rice (g/day)	251.8 ± 75.1	1.0 ± 3.4	<0.0001
Noodles (g/day)	19.4 ± 23.1	24.9 ± 30.4	0.027
Rice cakes (g/day)	2.6 ± 5.2	2.6 ± 8.9	0.994
Cereals (g/day)	0.05 ± 0.5	0.1 ± 1.1	0.615
Breads (g/day)	8.1 ± 18.0	8.3 ± 20.2	0.909
Sweet foods (g/day)	3.1 ± 8.0	3.5 ± 9.4	0.564
Nuts (g/day)	1.0 ± 3.3	0.6 ± 1.9	0.075
Beans (g/day)	22.7 ± 28.9	23.8 ± 29.8	0.664
Eggs (g/day)	9.3 ± 17.3	11.0 ± 19.9	0.309
Potatoes (g/day)	18.8 ± 25.6	14.6 ± 27.5	0.065
Salty vegetables (g/day)	154.6 ± 114.3	161.8 ± 106.0	0.455
Vegetables (g/day)	63.4 ± 58.2	60.1 ± 62.3	0.516
Meats (g/day)	16.2 ± 24.6	20.0 ± 24.4	0.073
Soups (g/day)	4.0 ± 10.2	3.6 ± 6.6	0.554
Fish (g/day)	43.9 ± 55.0	34.8 ± 44.1	0.025
Seafood (g/day)	4.5 ± 8.2	6.5 ± 14.5	0.090
Dairy products (g/day)	92.8 ± 127.4	67.7 ± 113.1	0.019
Soymilk (g/day)	17.7 ± 45.9	27.2 ± 83.8	0.149
Coffee (g/day)	8.2 ± 8.1	10.5 ± 9.2	0.004
Green tea (g/day)	22.9 ± 44.6	20.9 ± 46.4	0.605
Soft drinks (g/day)	21.1 ± 40.9	24.2 ± 45.7	0.388
Fruits & fruit juices (g/day)	172.6 ± 155.8	135.4 ± 116.3	0.001

MFDF: Multigrain rice, Fish, Dairy products, and Fruits & fruit juices; WNC: White rice, Noodles, and Coffee; Values are the Mean ± SD; ^1^ *t*-test for continuous variables.

General characteristics of the participants by dietary pattern are shown in Table 2. A total of 589 participants (77.0%) were classified into the MFDF dietary pattern, while 176 participants (23.0%) were classified into the WNC dietary pattern. Mean ages of the MFDF and WNC dietary patterns were 67.5 (SD = 5.0) years and 67.9 (SD = 5.7) years, respectively. The proportion of men was significantly higher in the WNC dietary pattern (60.2%, *p* < 0.0001). There were no significant differences in the proportions of educational levels between the two dietary patterns. “Farmer” was the major job of the participants in both dietary patterns. Significant differences existed between the two dietary patterns in regards to current alcohol drinkers, regular exercise, and diabetes. The proportion of current alcohol drinkers was higher in the WNC dietary pattern than the MFDF dietary pattern (*p* = 0.001). Conversely, the proportions of those who exercised regularly (*p* < 0.0001) and were diagnosed with diabetes (*p* = 0.002) were higher in the MFDF dietary pattern than the WNC dietary pattern. No significant differences in age, BMI (Body Mass Index), educational level, occupation, marital status, smoking status, dietary supplement use, or medical history, except for diabetes, were found between the two dietary patterns.

**Table 2:** General characteristics of the participants in the two dietary patterns.

Characteristics	MFDF (*n* = 589)	WNC (*n* = 176)	*p* ^1^
Age (years)	67.5 ± 5.0 ^2^	67.9 ± 5.7	0.311
Height (cm)	156.3 ± 8.4	158.2 ± 8.6	0.008
Weight (kg)	59.8 ± 9.3	60.3 ± 9.0	0.513
BMI	24.5 ± 3.2	24.1 ± 3.1	0.172
Men (%)	38.2	60.2	<0.0001
Education (%)			0.355
Uneducated	17.8	22.9	
Elementary	53.1	45.7	
Middle school	12.2	12.6	
High school	11.4	14.3	
College or Higher	5.4	4.6	
Occupation			0.279
Office work	5.3	7.4	
Non-office work	2.0	1.7	
Service industry	4.6	4.0	
Farmer	52.8	56.8	
Housework	13.8	8.5	
Unemployed	19.7	17.6	
Others	1.9	4.0	
Marital status			0.226
Currently married, or cohabiting (%)	78.8	83.0	
Alone (%)	21.2	17.1	
Current alcohol drinker (%)	36.8	50.6	0.001
Current smoker (%)	2.2	4.0	0.197
Regular exerciser (%)	34.1	15.3	<0.0001
Dietary supplement user (%)	12.1	8.0	0.129
Disease			
Cardiovascular disease (%)	7.1	5.7	0.503
Hypertension (%)	36.0	28.4	0.063
Hyperlipidemia (%)	3.2	2.3	0.516
Diabetes (%)	13.9	5.1	0.002
Stroke (%)	2.0	3.4	0.292

MFDF: Multigrain rice, Fish, Dairy products, and Fruits & fruit juices; WNC: White rice, Noodles, and Coffee; BMI: Body Mass Index; ^1^ *t*-test for continuous variables and Chi-square test for categorical variables; ^2^ Mean ± SD.

Dietary intakes of the participants are presented in Table 3 after adjusting for sex. The total energy intake was not different between the two dietary patterns, but the mean percentages of energy intake from carbohydrate, protein, and fat were significantly different. No significant differences in carbohydrate intake were observed between the two groups. However, protein and fat consumption were higher in the MFDF dietary pattern than the WNC dietary pattern. Consumption of β-carotene and B vitamins (vitamin_6_ and folate) was also higher in the MFDF dietary pattern. The same tendency was observed for *n*-3 polyunsaturated fatty acid. The consumptions of potassium and calcium were significantly higher in the MFDF dietary pattern. There were no significant differences in the intakes of sodium, magnesium, iron, zinc, and selenium between the two dietary patterns. The MAR score, representing diet quality, was also higher in the MFDF dietary pattern than in the WNC dietary pattern.

**Figure 1:**
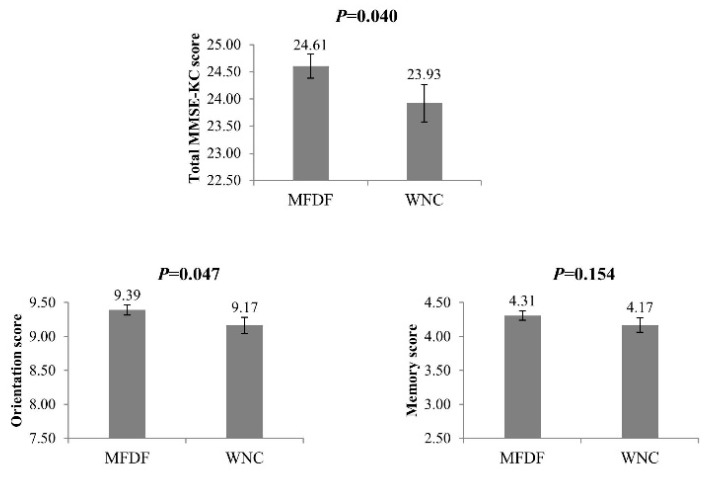
Means of the total MMSE-KC, orientation, and memory scores across the two dietary patterns. MFDF: Multigrain rice, Fish, Dairy products, and Fruits & fruit juices; WNC: White rice, Noodles, and Coffee. All dietary patterns were adjusted for sex, alcohol consumption, regular exercise, and diabetes.

Analysis of the MMSE-KC scores revealed significant differences between the MFDF and the WNC dietary patterns. The mean of the total MMSE-KC score of the participants in the MFDF dietary pattern was higher than that of the WNC dietary pattern after adjusting for sex, alcohol consumption, regular exercise, and diabetes (*p* = 0.040). In addition, the mean of orientation score from the MMSE-KC was also higher in the MFDF group after adjusting for sex, alcohol consumption, regular exercise, and diabetes (*p* = 0.047). (Figure 1).

The association between the dietary patterns and cognitive impairment was explored through logistic regression analysis (Table 4). Compared with the WNC dietary pattern, the MFDF dietary pattern showed lower risk of cognitive impairment after adjusting for age, sex, educational level, alcohol consumption, regular exercise, and diabetes (OR 0.64, 95% CI 0.44–0.94).

**Table 3:** Nutrient intake of participants in the two dietary patterns.

Dietary intakes	MFDF (*n* = 589)	WNC (*n* = 176)	*p* ^1^
Total energy (kcal)	1464.6 ± 18.2 ^2^	1422.9 ± 32.7	0.270
Percentage of energy			
From carbohydrate (%)	76.1 ± 0.3	78.1 ± 0.5	0.0002
From protein (%)	11.8 ± 0.1	11.1 ± 0.2	<0.0001
From fat (%)	12.4 ± 0.2	10.3 ± 0.4	0.0003
Carbohydrate (g)	275.9 ± 3.2	274.4 ± 5.7	0.815
Protein (g)	46.1 ± 0.8	40.3 ± 1.4	0.0004
Fat (g)	20.2 ± 0.5	17.5 ± 1.0	0.015
Total fatty acid (g)	10.1 ± 0.3	9.0 ± 0.6	0.102
Saturated fatty acid (g)	4.3 ± 0.2	3.8 ± 0.3	0.151
Monounsaturated fatty acid (g)	4.5 ± 0.2	4.1 ± 0.3	0.269
Polyunsaturated fatty acid (g)	2.4 ± 0.1	2.1 ± 0.1	0.091
*n*-3 polyunsaturated fatty acid (g)	0.4 ± 0.02	0.3 ± 0.03	0.040
β-carotene (μg)	2567.9 ± 79.9	2219.8 ± 143.8	0.036
Vitamin C (mg)	78.9 ± 2.1	71.8 ± 3.8	0.104
Vitamin E (mg)	6.6 ± 0.1	6.1 ± 0.3	0.115
Vitamin D (μg)	2.0 ± 0.1	1.7 ± 0.2	0.051
Vitamin B_6_(mg)	1.2 ± 0.02	1.0 ± 0.03	0.001
Vitamin B_12_(μg)	2.2 ± 0.1	2.2 ± 0.2	0.837
Folate (μg)	417.0 ± 7.1	363.2 ± 12.8	0.0003
Sodium	2861.4 ± 68.0	2806.4 ± 122.3	0.697
Potassium	2072.6 ± 39.1	1754.3 ± 70.3	<0.0001
Calcium	368.8 ± 9.7	302.8 ± 17.4	0.001
Magnesium	39.7 ± 1.1	35.1 ± 2.0	0.051
Iron(mg)	37.4 ± 2.3	33.8 ± 4.2	0.455
Zinc(mg)	7.9 ± 0.1	7.5 ± 0.2	0.197
Selenium(μg)	64.8 ± 1.0	64.0 ± 1.8	0.703
MAR	0.7 ± 0.01	0.6 ± 0.01	<0.0001

MFDF: Multigrain rice, Fish, Dairy products, and Fruits & fruit juices; WNC: White rice, Noodles, and Coffee; MAR: mean adequacy ratio; ^1^ *p* values for differences across two dietary patterns were obtained using the general linear model after adjusting for sex; ^2^ Least Squares Mean ± SE.

**Table 4:** Odds ratio with 95% confidence interval for cognitive impairment according to dietary patterns.

	Model 1 ^1^	Model 2 ^2^	Model 3 ^3^
WNC	1.00(reference)	1.00	1.00
MFDF	0.67 (0.47, 0.95)	0.62 (0.43, 0.90)	0.64 (0.44, 0.94)

MFDF: Multigrain rice, Fish, Dairy products, and Fruits & fruit juices; WNC: White rice, Noodles, and Coffee; ^1^ Model 1 was not adjusted; ^2^ Model 2 was adjusted by age, sex, and education; ^3^ Model 3 was adjusted by age, sex, education, alcohol consumption, regular exercise, and diabetes.

## 4. Discussion

Two distinct dietary patterns were identified by cluster analysis in older Korean adults residing in rural areas. The participants (77.0%) with high consumption of multigrain rice, fish, dairy foods, and fruits and fruit juices were assigned to the MFDF dietary pattern, while the remaining 23.0% with higher intakes of white rice, noodles, and coffee were assigned to the WNC dietary pattern. The MFDF pattern was associated with lower risk of cognitive impairment than the WNC dietary pattern.

Several studies have reported the association between chronic diseases and dietary pattern [30,31,32], but limited studies have been done on the relationship between dietary patterns and cognitive function. Among some of the studies reporting relations between dietary patterns and cognitive function, the “healthy” dietary pattern reported in a middle-aged French population [15] and “whole food” dietary pattern in Australians aged 65 years of age or over [18] were similar to the MFDF dietary pattern observed in this study. No similar pattern to the WNC dietary pattern was present among Western studies.

The MFDF and WNC dietary patterns were similar to the dietary patterns of previous studies carried out among Koreans [33,34,35]. The MFDF dietary pattern (high consumption of multigrain rice, fish, dairy products, and fruits and fruit juices) was generally similar to the “Korean Healthy” dietary pattern (higher consumption of whole grains, legumes, vegetables, and fruits) [34] and the “modified traditional” dietary pattern (lower consumption of white rice and higher consumption of fruits, dairy products, and legumes) [33]. On the other hand, the WNC dietary pattern was analogous to the “Traditional Korean” dietary pattern (higher consumption of white rice and lower consumption of milk products) [35], “Rice-oriented” dietary pattern (higher consumption of white rice and lower consumption of vegetables, fruits, meat, and dairy products) [36], and “Traditional” dietary pattern (higher consumption of white rice) [33].

The proportion of participants who exercised regularly was higher in the MFDF dietary pattern than in the WNC dietary pattern. Recent research has identified physical activity as a potential protective factor for preventing mild cognitive impairment [37,38,39]. When adults experiencing subjective memory impairment engaged in regular physical activity through a six-month program, a modest improvement in cognition was observed [37]. Moreover, meta-analysis concluded that aerobic activities are beneficial for cognition in healthy older adults [38].

A major staple food in the Korean diet is rice, which accounts for 32.7% of total calorie intake [40]. Koreans eat plain white rice, or rice mixed with multigrain (brown rice, black rice, barley, or millet *etc*.) and/or beans. White rice and brown rice have similar amounts of calories, but white rice consists mostly of starchy endosperm because a great portion of the nutrients of brown rice is eliminated during the polishing process. Several vitamins and minerals such as B vitamins (B_1_, B_2_, and B_6_), α-tocopherol, vitamin E, iron, zinc, and selenium are lost through the polishing process. Thus, mixed-grain rice contains more B vitamins (B_1_, B_2_, and B_6_), α-tocopherol, vitamin E, iron, zinc, and selenium than white rice. The constituents of multigrain rice, such as B vitamins, α-tocopherol, vitamin E, iron, zinc, and selenium, are thought to exert favorable effects on cognitive function [41]. However, to our knowledge, no study has directly examined the relationship between multigrain intake and cognitive function, apart from a constituent of a dietary pattern. Therefore, further studies on the relationship between whole grain intake and cognitive function are required.

The MFDF dietary pattern showed higher intakes of multigrain rice, fish, dairy products, and fruits and fruit juices. Such foods can be good dietary sources of antioxidant vitamins, B-vitamins, and *n*-3 polyunsaturated fatty acid. So far, the evidence from observational studies is insufficient to exert a definitive association between several nutrients and cognitive function [12,13,42]; however, several studies have supported the beneficial effects of higher intakes of β-carotene, B-vitamins, and n-3 fatty acids for cognitive function [11,43,44]. Fish is a good source of n-3 polyunsaturated fatty acids, which are major constituents of nerve cell membranes and have anti-inflammatory effects, possibly providing support for their protective effects against cognitive impairment [45]. B-vitamins (Vitamin B_6_ and folate) can be consumed through whole grains and fruits. Many studies have reported a significant association between hyperhomocysteinemia and cognitive impairment [46]. Deficiency of folate, Vitamin B_6_ or B_12_ is a major factor influencing homocysteine (Hcy) concentration [46,47]. Fruit is rich in antioxidant vitamins. In the present study, participants of the MFDF dietary pattern consumed more β-carotene compared to those demonstrating the WNC dietary pattern. β-carotene plasma levels were reported to be related to better memory performance [48]. A randomized trial reported that long-term β-carotene supplementation for 18 years may be necessary to obtain cognitive benefits [43]. Brain tissue contains easily oxidizable fatty acids which require the protection of fat-soluble antioxidant vitamins such as β-carotene. Adequate intake of β-carotene may protect brain tissue against oxidative damage by free radicals. Therefore, the sufficient intake of *n*-3 polyunsaturated fatty acids, B-vitamins, and β-carotene in the MFDF dietary pattern may explain the lower risk of cognitive impairment.

In this study, potassium and calcium intakes were significantly higher in the MFDF dietary pattern compared to the WNC dietary pattern. Fruits are rich in potassium and dairy products are a good source of calcium. Ozawa M *et al*., reported that potassium and calcium intakes reduced the risk of vascular dementia [49]. However, Cherbuin N *et al*. observed that a higher intake of potassium was related to an increased risk of developing MCI and that there was no effect of calcium on MCI [50]. Further studies on the effects of potassium and calcium intakes on cognitive function are necessary.

MAR evaluates overall dietary quality. The MFDF dietary pattern showed a higher MAR score than the WNC dietary pattern after adjusting for sex. Several studies have reported that consumption of a healthy diet, defined by *a priori* hypotheses such as the Mediterranean diet (MeDi) score, the Healthy Eating Index (HEI), and the Recommended Food Score (RFS), was associated with better cognitive function among the elderly [51,52,53,54,55,56,57]. Higher quality diets may help to protect against age-related cognitive decline in the elderly.

The WNC dietary pattern, centered on staple foods such as white rice and noodles without the consumption of various foods, was identified in this study. This pattern was associated with lower cognitive function. Previous studies have reported that unbalanced dietary patterns such as “white rice, kimchi, and seaweed” [58], “white rice and kimchi” [59], and “rice-oriented” [36] dietary patterns were related to health problems in Koreans. The “White rice, kimchi, and seaweed” dietary pattern was negatively related to bone health [58], the “white rice and kimchi” dietary pattern was positively related to obesity [59], and the “rice-oriented” dietary pattern was positively related to hypertriglyceridemia and low high density lipoprotein-cholesterol [36]. Therefore, white rice-centered unbalanced dietary patterns could influence the development of health conditions. In the WNC dietary pattern, coffee intake was higher than for the MFDF dietary pattern. Some studies reported that caffeine could be helpful for preventing cognitive impairment and dementia [60]. Although the intake of caffeinated drinks such as coffee was higher in the WNC dietary pattern, it seems difficult to show beneficial effects of caffeine alone without the consumption of healthy foods.

Limitations of this study should be noted when interpreting the results. Since this was a cross-sectional study, we cannot conclude causality of dietary patterns for cognitive impairment. Although we tried to reduce the influence of participants with lower cognition by eliminating the participants who fell in the lowest 5% of MMSE-KC scores, there is still the possibility of reverse causality. Because old adults with cognitive impairment may lose their ability to prepare adequate meals, cognitive impairment can affect dietary pattern. Second, a gold standard for determination of the number of clusters has not yet been established [61], and dietary pattern analysis (cluster analysis) requires the subjective decisions of the investigator. Third, depression status was not considered as a potential confounder because a small number of subjects were included in the depression survey. In addition, although we tried to control for covariates, except for depression status, it is possible that there were residual confounders influencing cognitive function, such as unknown risks or protective factors. Despite these limitations, the dietary patterns identified in this study were similar to the previous studies on Koreans [33,34], and the result of previous studies which suggested adhering to a healthy diet could lower the risk of cognitive impairment was also confirmed [15,18].

## 5. Conclusions

In conclusion, this study identified two distinct dietary patterns, the MFDF dietary pattern and the WNC dietary pattern. The MFDF dietary pattern, consisting of higher intakes of multigrain rice, fish, dairy products, and fruits and fruit juices, showed lower risk of cognitive impairment among older Korean adults than the WNC dietary pattern, which was made up of higher intakes of white rice, noodles, and coffee. The present study demonstrated that dietary pattern was related to the risk of cognitive impairment among Korean older adults. However, since this was a cross-sectional study, further investigations are necessary to identify causal associations between dietary pattern and cognitive impairment among Korean older adults.
